# Dietary Fat in Relation to Erythrocyte Fatty Acid Composition in Men

**DOI:** 10.1007/s11745-013-3832-0

**Published:** 2013-08-24

**Authors:** Markus Takkunen, Jyrki Ågren, Johanna Kuusisto, Markku Laakso, Matti Uusitupa, Ursula Schwab

**Affiliations:** 1 Institute of Public Health and Clinical Nutrition, University of Eastern Finland, P.O. Box 1627, 70211 Kuopio, Finland; 2 Institute of Biomedicine, University of Eastern Finland, Kuopio, Finland; 3 Department of Medicine and Kuopio University Hospital, University of Eastern Finland, Kuopio, Finland; 4 Institute of Public Health and Clinical Nutrition, University of Eastern Finland, and Research Unit, Kuopio University Hospital, Kuopio, Finland; 5 Institute of Public Health and Clinical Nutrition, University of Eastern Finland, and Institute of Clinical Medicine, Internal Medicine, Kuopio University Hospital, Kuopio, Finland

**Keywords:** Dietary fatty acids, Erythrocyte phospholipids, Food frequency questionnaire, Fat quality

## Abstract

Erythrocyte membrane fatty acid (EMFA) composition is used in the validation of food frequency questionnaires (FFQ) and the evaluation of dietary fat quality. In this cross-sectional study we aimed to investigate associations of diet with EMFA. Altogether, 1,033 randomly selected Finnish men, aged from 47 to 75 years filled in a FFQ and their EMFA composition was analyzed. Marine polyunsaturated fatty acid (PUFA) intake correlated positively with erythrocyte eicosapentaenoic and docosahexaenoic acids (*r*_s_ = 0.415 and *r*_s_ = 0.340, respectively, *P* < 0.001) and inversely with all n-6 PUFA analyzed (*P* < 0.001). PUFA intake from spreads and cooking fats correlated positively with alpha-linolenic (ALA), linoleic (LNA) and nervonic acids (*r*_s_ = 0.229, *r*_s_ = 0.160 and *r*_s_ = 0.143, respectively, *P* < 0.001). Milk fat intake was associated with myristic and behenic acids (*r*_s_ = 0.186 and *r*_s_ = 0.132, respectively *P* < 0.001). Butter users had lower ALA and LNA proportions (mol%) than non-users (0.16 ± 0.04 vs. 0.19 ± 0.05, *P* < 0.001 and 7.77 ± 1.02 vs. 8.12 ± 1.11, *P* = 0.001). Higher PUFA intake from meat was related to decreased long-chain n-3 (*P* < 0.001) and increased n-6 PUFA (*P* < 0.001) proportions. In conclusion, EMFA composition reflects particularly well the intakes of n-3 PUFA, whereas other associations remained lower. Yet, all main sources of dietary fat were related with EMFA. The dietary effect on the nervonic acid proportion was confirmed.

## Introduction

Fatty acids (FA) incorporated as phospholipids in red blood cell membranes are commonly used in the validation of dietary questionnaires and the assessment of dietary fat quality. Erythrocyte membrane fatty acids (EMFA), as well as FA in other lipid fractions (e.g. cholesterol esters and phospholipids in plasma), are seen as unbiased biomarkers of dietary fat quality in comparison to dietary questionnaires. FA profiles of different lipid fractions vary and EMFA are characterized by, to list a few, abundant proportions of palmitic (16:0), stearic (18:0) and arachidonic acids (ARA, 20:4n-6) and a relatively low linoleic acid (LNA, 18:2n-6) proportion [1, 2]. Proportions of FA are varyingly interrelated among blood lipids and changes in them after dietary intervention are to some extent different [1, 3, 4]. Erythrocytes have lifespan of several months and lack enzymes needed for FA metabolism, thus EMFA are thought to represent diet within the past months, i.e. longer than the FA of plasma, and could therefore be a better biomarker of long-term fat intake than plasma FA [5]. However, this claim remains controversial [1]. Furthermore, it is important to note that a large part of variation in erythrocyte membrane PUFA composition seems to be explained by genetic factors [6–10].

The intake of marine long-chain n-3 polyunsaturated fatty acids (PUFA) correlates especially well with proportions of eicosapentaenoic acid (EPA, 20:5n-3) and docosahexaenoic acid (DHA, 22:6n-3) in observational studies using EMFA [5, 6, 11]. Similarly, erythrocyte LNA is associated with dietary LNA intake [3, 5, 12–14]. High inverse correlation between marine PUFA intake and erythrocyte ARA has also been reported [15]. Even though the proportion of ARA in EMFA is modified already by a low dose of ARA supplementation [16], virtually no positive associations with proportions of ARA have been reported in observational studies [1]. In a few studies some saturated (SFA) and monounsaturated (MUFA) fatty acids (e.g. myristic and oleic acids, respectively), as well as alpha-linolenic acid (ALA, 18:3n3), have been shown to be associated with dietary intake [3, 5, 11, 13]. In general, these associations are substantially weaker or nonexistent compared with those of marine PUFA. Moreover, very little information on dietary determinants of long-chain SFA and MUFA typical for sphingomyelin (e.g. nervonic acid [24:1n-9]) is available.

There have also been studies assessing relationship between EMFA and chronic diseases. Of note are EPA and DHA, of which proportions in erythrocytes have been associated with cardiovascular morbidity [17–19], depression [20], social anxiety disorder [21], atopy in children [22], the sum of EPA and DHA has been proposed to be a risk marker for coronary heart disease [23], and in whole blood have also been connected to mortality [24, 25]. There have been fewer associations of other EMFA with different diseases and some results have been inconclusive. For example, higher erythrocyte ALA has been associated with cardiac arrest [26] but lower ALA was connected to ischemic stroke in one study [19]. Erythrocyte LNA was inversely associated with acute coronary syndrome [27], which is supported by the fact that dietary intake of LNA is acknowledged to have beneficial health effects [28]. Furthermore, some erythrocyte SFA and MUFA were associated with sudden cardiac arrest [29] or development of type 2 diabetes [3, 30], and recently palmitoleic acid (16:1n-7) has been associated with metabolic syndrome [31].

In this study we investigated how different dietary patterns are related to EMFA composition using a cross-sectional approach. For this purpose a food frequency questionnaire (FFQ) and EMFA data from 1,033 middle-aged and elderly men were analyzed. Knowing that dietary habits change, and that for some reason EMFA have been used less frequently in large observational studies than fractions of plasma [1], we brought extensive, up-to-date information on how FA from main fat sources modulate FA composition of red blood cell membranes in this large cohort of Finnish men.

## Subjects and Methods

### Study Population

Originally, a total of 10,197 men, aged from 45 to 70 years, participated in the cross-sectional Metabolic Syndrome in Men study (METSIM) in 2005–2010. The study population was selected randomly from the population register of the city of Kuopio (population 97,000) located in eastern Finland. The study protocol has been reported earlier [32]. The subjects were invited to an ongoing follow-up study (follow-up time an average 5 years). A subsample of over 1,000 men, aged from 47 to 75 years, who participated consecutively in a follow-up examination in 2010–2011 were asked to complete a FFQ and a blood sample was drawn to measure the EMFA composition. The final number of subjects in each statistical model and figure ranged between 904 and 1,012 depending on availability of complete data.

The METSIM study was approved by the Ethics Committee of the University of Kuopio and Kuopio University Hospital (17/2004) and was conducted in accordance with the Helsinki Declaration. All participants gave written informed consent.

### Clinical and Laboratory Measurements

The subjects had a 1-day outpatient visit to the Clinical Research Center at the University of Eastern Finland where clinical measurements were completed and blood samples were drawn after an overnight fast. Height was measured (without shoes) in the Frankfurt position to the nearest 0.5 cm. Weight was measured in light clothing with a digital scale (Seca 877, Seca, Hamburg, Germany), and rounded to the nearest 0.5 kg. The body mass index was calculated as weight divided by height in meters squared (kg/m^2^). The subjects were also asked about their smoking status, alcohol consumption and medication.

### Erythrocyte Membrane Fatty Acid Composition Analysis

Erythrocytes were separated from EDTA-blood by centrifugation at 1,000*g* for 10 min (4 °C) and hemolyzed in the tris–HCl buffer (pH 7.6, 10 mmol/l). Erythrocyte membranes were prepared by centrifugation of hemolysate at 30,000*g* for 30 min at 4 °C. Membrane sediment was resuspended in 0.5 ml of distilled water. FA methyl esters were prepared by direct transesterification, which gives more complete recovery especially of sphingomyelin-derived FA compared to separate extraction and transesterification [33]. Briefly, after mixing 0.1 ml of membrane suspension and 2 ml of methanol–toluene (4:1, v:v) in a glass tube, 0.2 ml of acetyl chloride was slowly added and this mixture was incubated at 100 °C for 1 h. After cooling in cold water 5 ml of 6 % K_2_CO_3_ was carefully added and then vigorously shaken. Toluene was separated into an upper phase by centrifugation at 2,000*g* for 5 min and injected into the gas chromatograph (Agilent Technologies 7890A) with a 25-m FFAP column (Agilent Technologies, Wilmington, DE). Pure standards (NU Chek Prep, Inc., Elysian, MA) were used for the identification of FA methyl esters and for the preparation of calibration curves. Heptadecanoic acid methyl ester (17:0) served as an internal standard. The intra and inter-assay precision varied between 0.3 and 4.1 % and between 2.0 and 9.1 % (relative standard deviation), respectively.

### Food Frequency Questionnaire

We used a FFQ similar to the one which had been previously used in the FINRISK study [34]. There were only minor changes in the FFQ used. In our study the information about fish species and dairy products consumed was inquired after in more detail, bread type classifications were more specified and the brand names for spreads and cooking fats were updated (see Table 1).

**Table 1:** The formation of fat intake variables for specific food groups

Food group	Products in the FFQ	Created variables	Weighted by	*n* ^a^
Fish	Oily fishes (e.g. rainbow trout), Baltic herring, freshwater fishes with medium fat content (e.g. vendace), low fat fishes (e.g. perch), fish oil supplement	PUFA	FA	983
Milk and milk products	Milk, hot chocolate, sour milk (fat > 1 %), unflavored yoghurt (fat > 1 %), flavored yoghurt (fat > 1 %), cheese (fat ≤ 17 %), cheese (fat > 17 %), ice cream	SFA, MUFA^c^	FA, portion size, milk type	951
Meat	Meat (minced meat, beef etc.), sausages, cold cuts, cold cuts (full meat)	SFA^b^, MUFA^b^, PUFA	FA, portion size	1,012
Spreads and cooking fat	Spread, cooking fat	SFA, MUFA^b^, PUFA	FA, portion size, bread slices	967
Butter use	Spread, cooking fat	Yes/No	–	122/871
Fast food	Pizza, hamburgers, French fries, ready meals, nibbles (potato chips, popcorn)	Overall	–	977

*FA* fatty acid, *FFQ* food frequency questionnaire, *MUFA* monounsaturated fatty acid, *PUFA* polyunsaturated fatty acid, *SFA* saturated fatty acid

^a^ FFQ and erythrocyte fatty acid composition data available

^b^ Variables were omitted from the analyses due to high intercorrelation (*r*_s_ > 0.90) with PUFA variable in the same food group

^c^ A variable was omitted from the analyses due to high intercorrelation (*r*_s_ > 0.90) with SFA variable in the same food group

During their visit to the research center, a total of 1,033 participants filled in the FFQ. They were asked to choose from eight frequency categories (ranging from never/less than once a month to four or more frequently on one day) how often they had used specified food products during the past 12 months. Bread consumption was asked with three questions in which the subjects chose how many slices they eat from eight categories (never/slice a week to six or more slices a day). The subjects were also allowed to freely specify how many glasses (2 dl) of milk, hot chocolate and sour milk they drink. In subsequent questions milk, spread and cooking fat types usually consumed were inquired. Lastly, the participants were asked which nutrient supplements they had used during the previous 6 months.

The FFQ results were used to create continuous combination variables which were presumed to relate to different levels of FA intakes. Four questions concerning fish types consumed were combined by summing up the frequencies transformed into times a day which were then multiplied by PUFA content of different fish types (e.g. twice a day multiplied by 2.6 g per 100 g of rainbow trout or salmon). The use of fish oil supplement was included in this sum of fish-derived PUFA intake (approximation of 0.65 g/day of PUFA was used). For milk and milk products SFA and MUFA content was calculated similarly. For different milk products, estimations of portion sizes were used (e.g. cheese 10 g per portion, yoghurt 150 g per portion). SFA, MUFA and PUFA intakes from meat were correspondingly calculated summing relevant variables weighted by portion size (e.g. cold cuts 20 g, meat 100 g) and FA content (e.g. meat PUFA 1.4 g/100 g). Additionally, SFA, MUFA and PUFA intakes from spreads and cooking fats were estimated by multiplying bread consumption by FA content of relevant spread (approximation of 5 g spread per slice was used) and summing this with cooking fat type (an approximation of 10 g/day was used for all types of cooking fats) weighted by its FA content. All SFA, MUFA and PUFA contents of different food products were derived from the Finnish Food Composition Database (Fineli ^®^, National Institute for Health and Welfare, Helsinki, Finland).

Since there were high intercorrelations (*r*_s_ > 0.90) within the food item groups, certain intake variables were omitted from the analyses (Table 1). Thus, the variable of SFA from milk and milk products closely reflects total SFA and MUFA intake from milk, the variable of PUFA from meat reflects total fat intake from meat and the variable of PUFA from spreads and cooking fat reflects MUFA and PUFA intake from spreads and cooking fat.

Finally, all these newly formed continuous variables were divided into the tertiles to reflect a more qualitative than quantitative scale of the FFQ. Additionally, total fast food consumption was estimated by summing unweighted frequencies per day of different types of fast food. Subjects with missing or unclear answers were excluded when the combination variables were formed. Valid numbers of subjects and specific information on how each variable was formed are shown in Table 1.

### Statistical Analysis

Values are presented means ± SD, if not otherwise stated. The fat intakes were divided into the tertiles in all statistical procedures except in the calculation of Spearman’s rank correlations with EMFA. Log-transformation was used to correct for skewness of variables, when appropriate. Differences between the means of continuous variables were tested by *t*-tests. ANCOVA was used to adjust for age, BMI, smoking, statin medication and alcohol consumption in the comparison of the groups, and coefficients of determination (*R* ^2^) were adjusted for the number of variables. Intake tertiles were forced as such into linear regression models adjusted for age, BMI, smoking, statin medication and alcohol consumption. Unstandardized linear regression coefficients are presented in the text (standardized coefficients are shown when dependents were log-transformed). All statistical analyses were performed using SPSS Statistics 19 (IBM, Armonk, New York).

## Results

The characteristics of 1,008 men who filled in the FFQ and had all other relevant data available are shown in Table 2.

**Table 2:** Characteristics of the study population (*n* = 1,008)

Variable	Value	
Age (years)	63	(59; 69)
Body mass index (kg/m^2^)	26.2	(24.2; 28.6)
Total alcohol consumption (g/week)	38	(0; 113)
HbA1c (%)^a^	5.7	(5.6; 6.0)
Fish oil supplement users (%)	22.9	
Smokers (%)	11.7	
Statin medication (%)	36.0	
Erythrocyte membrane fatty acids (mol%)		
14:0	0.46	(0.40; 0.51)
16:0	22.53	(21.81; 23.26)
18:0	15.36	(15.02; 15.74)
20:0	0.43	(0.39; 0.46)
22:0	1.78	(1.65; 1.93)
24:0	5.49	(5.19; 5.75)
16:1n-7	0.42	(0.35; 0.51)
18:1n-7	1.18	(1.11; 1.25)
18:1n-9	12.77	(12.19; 13.38)
20:1n-9 + 11	0.33	(0.30; 0.36)
24:1n-9	5.91	(5.56; 6.27)
18:3n-3	0.18	(0.15; 0.21)
20:5n-3	1.37	(1.08; 1.78)
22:5n-3	2.54	(2.30; 2.76)
22:6n-3	6.00	(5.26; 6.76)
18:2n-6	8.06	(7.39; 8.77)
20:3n-6	1.41	(1.25; 1.59)
20:4n-6	11.39	(10.68; 12.13)
22:4n-6	1.83	(1.58; 2.11)
22:5n-6	0.30	(0.25; 0.35)

Values are proportions or medians (25th percentile, 75th percentile)

^a^ *n* = 1,006

### Diet and Membrane Polyunsaturated Fatty Acids

Correlations between EMFA and dietary patterns are reported in Table 3. PUFA weighted fish and fish oil supplement intake was associated with erythrocyte membrane PUFA. The highest positive correlation was observed with EPA, whereas all n-6 PUFA were inversely associated with fish and fish oil supplement intake. PUFA weighted meat products were associated with higher ARA, adrenic acid (22:4n-6) and osbond acid (22:5n-6) proportions, whereas the correlations with EPA and DHA proportions were negative. Intake of spreads and cooking fat PUFA was associated with lower proportions of ARA and 22:5n-6 but with higher proportions of ALA and LNA. In addition, use of butter in cooking or on bread was related to lower proportions (mol%) of ALA and LNA (butter users 0.16 ± 0.04 vs. non-users 0.19 ± 0.05, *P* < 0.001 and 7.77 ± 1.02 vs. 8.12 ± 1.11, *P* = 0.001, respectively) but there was no difference in erythrocyte n-6/n-3 -ratio (2.34 ± 0.54 vs. 2.34 ± 0.51, *P* = 1.000).

**Table 3:** Spearman correlations between erythrocyte membrane fatty acids and diet weighted by fatty acid contents

Fatty acid	Saturated		Polyunsaturated		
	Milk and milk products	Spreads and cooking fat	Fish and fish oil supplements	Spreads and cooking fat	Meat products
14:0	0.186***	0.188***	–	−0.128***	–
16:0	–	–	–	–	–
18:0	–	–	−0.077	–	–
20:0	–	–	−0.070	–	–
22:0	0.132***	0.086*	−0.182***	−0.089*	–
24:0	–	–	–	–	–
16:1n-7	–	–	–	−0.182***	–
18:1n-7	–	–	–	–	–
18:1n-9	–	–	–	–	–
20:1n-9 + 11	–	–	–	0.065	–
24:1n-9	−0.159***	−0.142***	–	0.143***	–
18:3n-3	–	–	–	0.229***	–
20:5n-3	−0.091*	–	0.415***	–	−0.136***
22:5n-3	–	–	0.141***	–	–
22:6n-3	–	–	0.340***	–	−0.127***
18:2n-6	0.104*	0.081	−0.121**	0.160***	–
20:3n-6	–	–	−0.149***	–	–
20:4n-6	–	–	−0.272***	−0.116**	0.160***
22:4n-6	–	–	−0.296***	–	0.128***
22:5n-6	0.099*	0.093*	−0.269***	−0.180***	0.132***

Spearman’s correlation coefficients were calculated between each erythrocyte fatty acid proportion and saturated or polyunsaturated fatty acid intake by food group taking into account approximated portion sizes. Only significant associations are shown (*P* < 0.05)

* *P* < 0.01, ** *P* < 0.001 and *** *P* < 0.0001

Next we analyzed the associations using linear regression models adjusted for age, BMI, smoking, statin medication and alcohol consumption with tertiles of consumptions as explanatory variables. The adjusted results were similar to the unadjusted statistics. Spread and cooking fat PUFA were still associated with the proportions of ALA and LNA (*B* = 0.011 and *B* = 0.188, both *P* < 0.001). Additionally, spread and cooking fat PUFA were inversely associated with the proportion of ARA (*B* = −0.141, *P* < 0.001). Also an inverse relationship between milk and milk product SFA and the proportion of EPA was nominally significant after adjustments (for log-transformed: *β* = −0.069, *P* = 0.031).

**Fig. 1:**
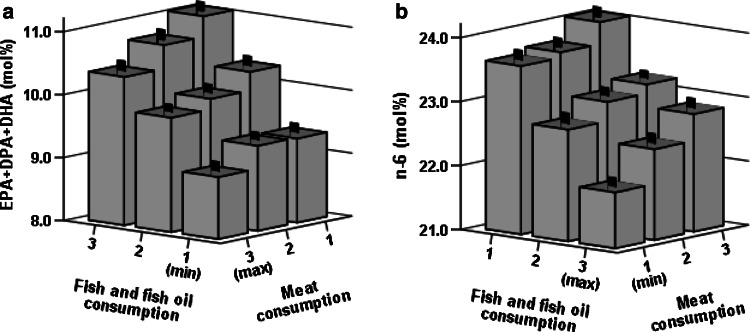
Values are means (mol%) and standard errors of erythrocyte membrane sums of EPA, DPA and DHA (**a**) and n-6 (**b**) in relation to PUFA weighted tertiles of intakes of fish and fish oil supplement and meat. In both figures *P* < 0.001 (*n* = 968) for both intake axes in age, BMI, smoking, statin medication and alcohol consumption adjusted ANCOVA models. 20.5 % of variation (*R* ^2^) in n-6/n-3 -ratio was explained by the model. Please note the differing order of categories on the horizontal axes. *DHA* 22:6n-3, *DPA* 22:5n-3, *EPA* 20:5n-3, *PUFA* polyunsaturated fatty acid

Figure 1a shows how the sum of marine derived PUFA (EPA, docosapentaenoic acid (DPA, 22:5n-3) and DHA) was positively related to PUFA intake from fish but inversely to PUFA intake from meat. There was a similar but opposite association with the sum of all measured n-6 PUFA (Fig. 1b). We did not find significant interactions between dietary fish and meat in relation to marine PUFA or n-6 PUFA in ANCOVA models adjusted for age, BMI, smoking, statin medication and alcohol consumption (*P*-interaction = 0.645 and *P*-interaction = 0.305, respectively).

### Diet and Membrane Saturated and Monounsaturated Fatty Acids

The proportions of SFA and MUFA in erythrocyte membranes were only weakly associated with diet (Table 3). Intake of dairy SFA was positively related to proportions of myristic acid (14:0) and behenic acid (22:0). The proportion of nervonic acid (24:1n-9) was associated inversely with SFA intake from milk and spreads and cooking fat, whereas there was a positive correlation between the proportion of nervonic acid and PUFA from spreads and cooking fat. The proportion of palmitoleic acid (16:1n-7) was also inversely related to PUFA from spreads and cooking fat. Similarly, the use of butter in cooking or on bread was associated with higher proportions (mol%) of myristic and behenic acids in erythrocytes (0.50 ± 0.08 vs. 0.45 ± 0.08, *P* < 0.001 and 1.82 ± 0.21 vs. 1.78 ± 0.21, *P* = 0.021) as well as with higher palmitoleic acid proportion (0.5 ± 0.15 vs. 0.44 ± 0.14, for log-transformation *P* < 0.001). Consistently, the proportion of nervonic acid was lower in butter users than non-users (5.71 ± 0.61 vs. 5.96 ± 0.53, *P* < 0.001).

**Fig. 2:**
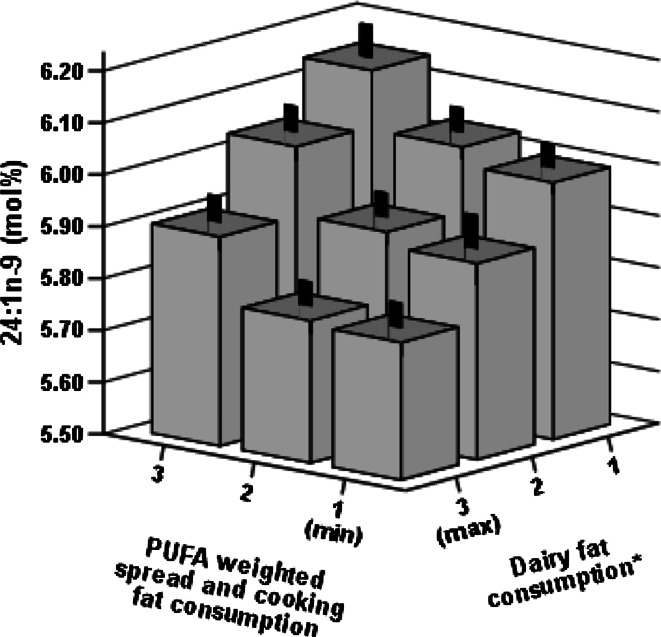
Values are means (mol%) and standard errors of the proportion of nervonic acid (24:1n-9) in the erythrocyte membrane in relation to diet (*n* = 904). Please note the differing order of categories on the horizontal axes. *Dairy fat consumption is the sum of saturated fatty acid weighted consumption of spreads and cooking fat and milk and milk products divided into tertiles

In adjusted linear regression models, the proportion of myristic acid remained associated with the intake of SFA from milk and spreads and cooking fat (*B* = 0.017 and *B* = 0.018, *P* < 0.001 for both). Similarly, the proportion of behenic acid was related to the intake of SFA from milk and spreads and cooking fat (*B* = 0.036, *P* < 0.001 and *B* = 0.020, *P* = 0.016) but inversely to the intake of marine PUFA (*B* = −0.041, *P* < 0.001). Furthermore, adding variables of spreads and cooking fat intake weighted by SFA and PUFA in the same regression model they were both still associated, in opposite directions, with the proportions of myristic (*B* = 0.018 and *B* = −0.011, both *P* < 0.001) and behenic acids (*B* = 0.021, *P* = 0.011 and *B* = −0.024, *P* = 0.003). Inverse association between the proportion palmitoleic acid and the PUFA intake from spreads and cooking fat also persisted (log-transformed: *β* = −0.158, *P* < 0.001). SFA from milk and milk products were negatively related to the proportion of nervonic acid (*B* = −0.108, *P* < 0.001). Adding the SFA and PUFA weighted spreads and cooking fat intake variables to the same regression model, both the SFA and PUFA variables were associated with the proportion of nervonic acid in opposite directions (*B* = −0.093 and *B* = 0.098, both *P* < 0.001). The opposite effect of SFA and PUFA on the proportion of nervonic acid is further illustrated in Fig. 2.

Consumption of fast food was uncommon (0.14 times a day) and associations remained very low (data not shown).

## Discussion

In agreement with many previous studies [3, 5, 6, 11, 13, 15], we observed the highest correlations between the proportions of marine PUFA in erythrocyte membranes (EPA and DHA) and marine PUFA intake. All other intake variables used in this study were related to EMFA composition, although less strongly. As a novel finding in an observational setting using EMFA, we report opposite effects of variables of SFA and PUFA on proportions of nervonic and behenic acids.

All erythrocyte n-6 FA were inversely associated with PUFA intake from marine sources, which is probably due to well-known competition between n-3 and n-6 for metabolic pathways [35]. We clearly demonstrated that the effects of fish and meat intakes on the proportions of n-3 and n-6 PUFA are additive, although opposite, and that marine PUFA intake is more strongly inversely associated with the proportion of n-6 PUFA than PUFA from meat. A significant effect of ARA intake on membrane phospholipids is rarely reported in observational studies. However, evidence from intervention studies is similar to our results showing that ARA intake increases proportions of ARA and adrenic acid (22:4n-6) in phospholipid membranes [16, 36, 37], and our results also showed an association with osbond acid (22:5n-6). In line with a Japanese study we found inverse associations between marine PUFA intake and the erythrocyte ARA proportion [15]. Furthermore, among pregnant Canadian women, there were decreased proportions of adrenic and osbond acids in erythrocyte ethanolamine phosphoglycerides related to higher dietary EPA and DHA intakes [38].

We also observed moderate to weak correlations between spreads and cooking fat and EMFA. Expectedly, erythrocyte ALA and LNA, which are high in vegetable oils (in Finland low erucic acid rapeseed oil is commonly used), correlated positively with PUFA weighted spreads and cooking fat intake, whereas use of butter was associated with lower proportions of ALA and LNA. Similar results have been reported in previous studies with LNA [3, 5, 13] and ALA [5, 11] in EMFA, although in some less recent studies correlations with LNA have been much higher [12, 14]. Spreads and cooking fat PUFA were also associated with lower ARA and osbond acid but not with EPA or DHA. It is known that ALA is a better substrate than LNA for Δ6 desaturase [39] and that the conversion of ALA to EPA, DPA and DHA is determined more likely by its absolute amount than by the ratio of ALA to LNA [40]. Thus our results suggest that ALA content of vegetable oil-based spreads and cooking oils consumed by the study population was high enough to prevent the decreasing effect of LNA on the proportions of EPA and DHA.

Positive correlations between diet and erythrocyte proportions of palmitoleic and myristic acids were reported in recent studies [3, 5]. This is explained by the fact that both myristic acid and palmitic acid can be desaturated into palmitoleic acid, and they are originating from the same sources, largely milk fat. In the present study, positive correlations were found between SFA weighted spreads and cooking fat and proportions of myristic and behenic acids along with the fact that butter users had a higher proportion of palmitoleic acid. Similarly, SFA weighted milk and milk products intake was associated with higher myristic and behenic acid proportions. Both of these intake variables correlated also negatively with nervonic acid proportion. Even though these two different variables of SFA intake were composed of entirely different sets of questions in the FFQ, their correlations with EMFA were strikingly similar and thus seem to represent the effect of dairy fats on EMFA composition.

The association between dairy fat and behenic acid is unlikely to be explained by its intake as there is only little behenic acid in dairy fat and it is poorly absorbed [41]. The more probable explanation is the increased production of behenic acid from shorter chain saturated FA present in dairy products. However, there was no association with lignoceric acid (24:0).

The influence of PUFA from cooking fat and spreads on SFA and MUFA was almost reciprocal to the effect of dairy fat, and there was actually a moderate negative correlation with palmitoleic acid. Even after both SFA and PUFA intakes from spreads and cooking fats were inputted to the same model, the opposite associations with myristic, behenic and nervonic acid persisted. It has to be noted that behenic and nervonic acids as well as lignoceric acid are specific for the sphingomyelin fraction of phospholipids, which should not be directly affected by PUFA. This and the fact that behenic acid was also negatively related to dietary marine PUFA, supports an indirect mechanism by which PUFA could suppress lipogenesis, elongases and stearoyl CoA desaturase, possibly by down regulating genes related to lipid metabolism [42].

Proportions of nervonic and lignoceric acids in rat liver sphingomyelin have been shown to be affected by their direct intake and to a lesser extent by their precursors [43]. In human platelets, borage oil, which contains some nervonic acid, was shown to decrease the proportion of behenic and increase the proportion of nervonic acid [44]. Thus, higher proportion of nervonic acid in the present study associated with PUFA from cooking fat and spreads is most likely explained by minute concentrations of nervonic acid and its immediate precursors 20:1n-9 and erucic acid (22:1n-9) in vegetable oils and margarines used by the Finns. This is also supported by a new large study, which related higher proportions of long-chain MUFA, including nervonic acid, in plasma phospholipids to the incidence of congestive heart failure and found several food products, such as poultry, affecting proportion of nervonic acid [45]. However, proportions of nervonic acid in plasma phospholipids and EMFA do not seem to be related [3] and, interestingly, a lower proportion of nervonic acid has been weakly associated with neurological diseases [46, 47].

We did not find any associations with oleic acid proportions, which is contrary to the high correlations reported among Italian women [13]. This is likely to be explained by lower olive oil consumption in Finland and demonstrates regional differences in EMFA and other lipid fractions [48]. Nevertheless, it is apparent that the type of spreads and cooking fat does not just influence the proportions of PUFA but also the proportions of SFA and MUFA are affected, even though the exact mechanisms, whether direct or indirect, are beyond the scope of this study.

There were also some significant, but low, associations of diet with EMFA not discussed above (e.g. negative correlation of milk fat with EPA), which are possibly explained by other differences in diet or lifestyle.

The precision of our food intake variables is hard to estimate, because significant associations with diet are typical for only some, mainly exogenous EMFA (e.g. EPA). Moreover, in the fully adjusted statistical model 20.5 % of variation in erythrocyte n-6/n-3 -ratio was explained by the explanatory variables, primarily by the variables of PUFA intake. Despite leaving the majority of variations unexplained, this still suggests that especially the variables of PUFA intake enable the assessment of fat intake and quality. Furthermore, Schaeffer et al. [10] have shown that genetic variations in desaturase coding genes *FADS1* and *FADS2* have a great impact on the variance of serum phospholipid PUFA composition, and this is true for EMFA as well [6–9].

This study has some limitations. Due to the FFQ used, the determination of exact FA intakes was impossible. This should not matter, since dietary intakes are often overestimated by FFQ compared with dietary history and this is also true for Finnish men [49]. Moreover, it might not always be sensible to calculate intakes, taking as an example, for EPA and DHA separately as their intakes are highly intercorrelated. Hence, our approach is more practical, providing direct information on how specific groups of foods affect EMFA.

We also lacked information on the total energy intake and total fat intake. Therefore, the variables of fat intake could not be adjusted by them. Instead, we adjusted for common confounders and the results were similar to the unadjusted statistics. After adjustments for confounders including BMI, alcohol consumption and age, it is unlikely that our results are explained solely by intakes of energy or total fat.

The strength of this study is the relatively large, regionally and ethnically homogenous, randomly sampled study population of middle-aged and elderly men. The lack of women makes the EMFA analysis easier since women are known to have some differences in FA metabolism compared with men [13, 50]. Besides, the use of EMFA and the FFQ together is very suitable, because both aim to reflect diet within past weeks and months.

In conclusion, we identified four distinct patterns between the main groups of dietary fat and EMFA. Of these the strongest and best known was the effect of marine PUFA that reflected as higher proportions of long-chain n-3 PUFA, whereas proportions of all n-6 PUFA were lower. PUFA intake from meat associated with higher proportions of long-chain n-6 and slightly lower n-3 PUFA in EMFA. Intake of PUFA from vegetable oils and spreads was associated, in a more complex manner, with higher proportions of ALA, LNA and nervonic acid but also lower proportions of some n-6 PUFA (e.g. ARA) and endogenously synthesized FA (e.g. behenic acid). Lastly, dairy fat associated with higher proportions of myristic and behenic acids and lower levels of nervonic acid, altogether this was almost a reciprocal effect compared with PUFA from spreads and cooking fats.

## Abbreviations

ALA: Alpha-linolenic acid
ARA: Arachidonic acid
DHA: Docosahexaenoic acid
DPA: Docosapentaenoic acid (n-3)
EMFA: Erythrocyte membrane fatty acid
EPA: Eicosapentaenoic acid
FA: Fatty acid(s)
FFQ: Food frequency questionnaire
LNA: Linoleic acid
METSIM: Metabolic syndrome in men-study
MUFA: Monounsaturated fatty acid(s)
PUFA: Polyunsaturated fatty acid(s)
SFA: Saturated fatty acid
